# Impact of COVID-19 on Adolescent HIV Prevention and Treatment Research in the AHISA Network

**DOI:** 10.1007/s10461-022-03811-5

**Published:** 2022-09-12

**Authors:** Elizabeth D. Lowenthal, Stephanie M. DeLong, Brian Zanoni, Irene Njuguna, Kristin Beima-Sofie, Dorothy Dow, Aisa Shayo, Alana Schreibman, Charisse V. Ahmed, Jennifer Chapman, Lydia Chen, Shreya Mehta, Michael T. Mbizvo

**Affiliations:** 1 Departments of Pediatrics and Biostatistics, Epidemiology and Informatics, University of Pennsylvania Perelman School of Medicine, Philadelphia, PA USA; 2 Children’s Hospital of Philadelphia Global Health Center, Philadelphia, USA; 3 Department of Epidemiology, Johns Hopkins Bloomberg School of Public Health, Baltimore, MD USA; 4 Departments of Medicine and Pediatric Infectious Diseases, Emory University School of Medicine, Atlanta, GA USA; 5 Children’s Healthcare of Atlanta, Atlanta, GA USA; 6 Kenyatta National Hospital, Research and Programs, Nairobi, Kenya; 7 Department of Global Health, University of Washington, Seattle, WA USA; 8 Department of Pediatrics, Duke University Medical Center, Durham, NC USA; 9 Duke Global Health Institute, Durham, NC USA; 10 Kilimanjaro Christian Medical Centre, Moshi, Tanzania; 11 Kilimanjaro Christian Medical University College, Moshi, Tanzania; 12 University of Pennsylvania, Philadelphia, PA USA; 13 University of Pennsylvania School of Nursing, Philadelphia, PA USA; 14 Population Council, Lusaka, Zambia; 15 CHOP Roberts Center for Pediatric Research, Room 11241, 734 Schuylkill Ave, Philadelphia, PA 19146 USA

**Keywords:** Pandemic, Africa, Implementation science, Survey

## Abstract

Members of the Adolescent HIV Prevention and Treatment Implementation Science Alliance (AHISA) network conduct research aiming to close gaps between what is known to be impactful across the HIV prevention and treatment cascade, and services delivered to optimize outcomes for adolescents/young adults (AYA) in high HIV-prevalence settings. The COVID-19 pandemic introduced new challenges which threaten to exacerbate care and access disparities. We report results of a survey among AHISA teams with active AYA HIV research programs in African countries to determine how the pandemic has impacted their efforts. Results highlighted the detrimental impact of the pandemic on research efforts and the expanded need for implementation research to help provide evidence-based, context-specific pandemic recovery support. Key lessons learned included the viability of remote service delivery strategies and other innovations, the need for adaptive systems that respond to evolving contextual needs, and the need for organized documentation plans, within empathic and flexible environments.

**Supplementary Information:**

The online version contains supplementary material available at 10.1007/s10461-022-03811-5.

## Introduction

The COVID-19 pandemic and related mitigation efforts (e.g., social distancing, stay at home restrictions) have impacted and will continue to influence HIV research globally, forcing researchers to both modify aspects of their studies and take into account additional contextual factors when conducting their research [1, 2]. The pandemic has led to adaptations to study protocols, recruitment materials, intervention delivery, and the staffing of research teams [3–5]. It has also raised new ethical requirements, including protecting study participants and research teams from COVID-19, while still conducting HIV research during a health crisis; and it has highlighted the fact that populations most impacted by HIV are often among those most negatively impacted by COVID-19 [2, 6]. Further, COVID-19’s impact on HIV programming and services, such as time between visits and refills, as well as viral load and CD4 count monitoring, have necessitated changes in how HIV prevention, treatment and care research study data are collected, analyzed and interpreted, the biases, including confounding, that need to be considered, and the overall generalizability of research study findings moving ahead [2, 7–10].

In some cases, the pandemic has necessitated expansions of the scope of ongoing HIV-focused research, has overextended HIV researchers, and has increased research funding needs. Besides the HIV prevention and treatment research already being conducted, additional HIV research has incorporated COVID-19, spanning clinical and laboratory assessments of COVID-19 among people living with HIV, the clinical impact of COVID-19 on HIV co-morbidities, and assessment of the pandemic’s impact on HIV services, providers, and prevention efforts [11–22]. Furthermore, multiple commentaries have been written drawing parallels of lessons learned from the HIV pandemic for application in the COVID-19 situation [23–25]. Several modeling studies carried out have also warned of increases in mortality owing to interruptions in HIV treatment [26–28].

For implementation science studies, another layer of complexity is added by the challenges of disentangling temporary COVID-related implementation barriers from implementation adaptations and innovations that are likely to endure post-pandemic. Rapid changes in HIV clinical care models necessitated by the pandemic have resulted in decreased routine data-capture, alterations in care continuum metrics, and the need for increased reliance on analytic measures that can help account for selection biases and measurement errors such as inverse probability weighting and multiple imputation[7]. However, so far, no structured evaluations of the impact of the pandemic on HIV implementation research have been published.

### Impact of COVID-19 on AYA HIV Research

Adolescents and young adults (AYA) living with HIV (ALHIV) remain a uniquely vulnerable group, with their HIV-related outcomes continuing to lag behind both older and younger age populations [29]. Youth aged 15–24 account for a large proportion of new HIV infections globally (around 30%), with an estimated 1.7 million adolescents aged 10–19 living with HIV worldwide, the majority in Africa [30, 31]. While AYA HIV incidence and mortality have declined in recent years [32], disparities remain and the COVID-19 pandemic threatens to reverse recent improvements. Despite the noted impacts of the COVID-19 pandemic on HIV research as a whole, how it has impacted AYA HIV prevention and treatment implementation research remains unclear.

The Adolescent HIV Prevention and Treatment Implementation Science Alliance (AHISA) is an U.S. National Institutes of Health (NIH)-supported network of AYA-focused HIV implementation science researchers and stakeholders who meet to further AYA HIV-focused prevention and treatment research in Africa [33]. AHISA researchers conducted a cross-network program evaluation survey to assess the impact of COVID-19 on AHISA teams’ research, programs, and clinical activities and to draw and share lessons learned early in the pandemic. Here, we share the research-related data from the AHISA network survey in order to help public health program managers, service delivery officials, policymakers, funders and fellow researchers understand the status of AYA HIV implementation research during the COVID-19 pandemic and how to best support AYA HIV prevention, care, and treatment research teams now and in the future.

## Methods

### Study Context, Design, and Processes

We conducted an AHISA network survey on the impacts of the COVID pandemic on research projects and clinical services related to HIV and AYA in our study settings. This involved a group of AHISA volunteers, who initiated the design and conduct of a membership survey to capture the impacts of COVID-19 on member teams and the research and clinical programs with which they are affiliated. AHISA member volunteers met virtually and agreed on volunteer roles, survey components and methods of seeking survey responses. Survey drafts were created and reviewed by all group members and additional trainee volunteers, were pilot tested among colleagues, and a consensus draft was programmed into REDCap electronic data capture tools hosted at the Children’s Hospital of Philadelphia for administration as an emailed survey [34, 35].

### Study Setting

AHISA teams are conducting 26 collaborative implementation research projects; specifically one team (4% of total) each in Botswana, Ghana, Malawi, and Rwanda; two teams (8%) each in Tanzania, Uganda, Zambia, and Zimbabwe; three teams (12%) in Nigeria; five teams (19%) in South Africa, and six teams (23%) in Kenya. The group of research teams represent East, West and Southern Africa. Most teams are fully distinct, but a few academic and public health leaders are members of more than one team. The foci of AYA-focused implementation research programs within the AHISA network span the HIV care continuum and also include studies focused on prevention, supportive disclosure of HIV status to younger adolescents, and effective transition of care from pediatric to adult programs.

### Data Collection

Survey links were emailed to the leader of each AHISA team in February 2021 and a single response from each team’s leader or a designee was requested. Follow-up requests to teams that did not respond to the initial survey continued through early April 2021.

The survey collected quantitative data (e.g. via multiple choice, Likert scale and numerical response options) and qualitative data (e.g. open-ended questions with longform written answers) related to research program and clinical service changes in response to the COVID-19 pandemic. We did not collect data specific to individuals with the exception of contact information for the responding team member to allow for clarification of survey responses. Surveys asked each team to indicate if they experienced interruptions in research recruitment and follow-up. For those who experienced interruptions, the duration and impacts of these interruptions was recorded. Teams were also asked to describe COVID-related research protocol modifications that were made, whether they introduced remote research activities, and whether their study funding was threatened by the pandemic. Each team was asked to provide text responses indicating what they thought were the three most important: (1) impacts of the COVID-19 pandemic on AYA-focused HIV research at their site, (2) adjustments or changes their team made due to the COVID-19 pandemic, and (3) lessons learned that they thought could be helpful to other teams conducting AYA HIV prevention and/or treatment research. Teams were also asked to respond to open-ended questions to gain a more detailed description of their experiences (e.g. their best example of an effective response to a COVID-19-related challenge). The full survey is included in Online Appendix 1. Only the research-related data are presented in this manuscript. Data related to clinical service impacts will be presented in a separate manuscript.

### Ethics

Prior to dissemination, plans for the survey were reviewed by a divisional review committee at the Children’s Hospital of Philadelphia and determined to be program evaluation without human subject data collection and exempt from IRB research oversight.

### Analysis

Counts with percentages were calculated for quantitative data related to the number of AHISA teams reporting pandemic-related impacts to their AYA HIV research activities. Open-ended responses to survey questions were coded in excel by a single investigator. Codes were developed inductively from the data to characterize the types of protocol modifications observed, challenges to study validity owing to the COVID-19 pandemic, and lessons learned. Qualitative data was organized thematically to describe similarities and differences between AHISA team experiences. Additional investigators reviewed the full dataset and suggested revisions to the data presentation. Findings are presented below. The authorship team synthesized data related to reported study design modifications and proposed solutions based both on innovations reported by the surveyed teams and the authors’ own ideas about potential responses to the reported challenges (See Table 3).

## Results

**Fig. 1:**
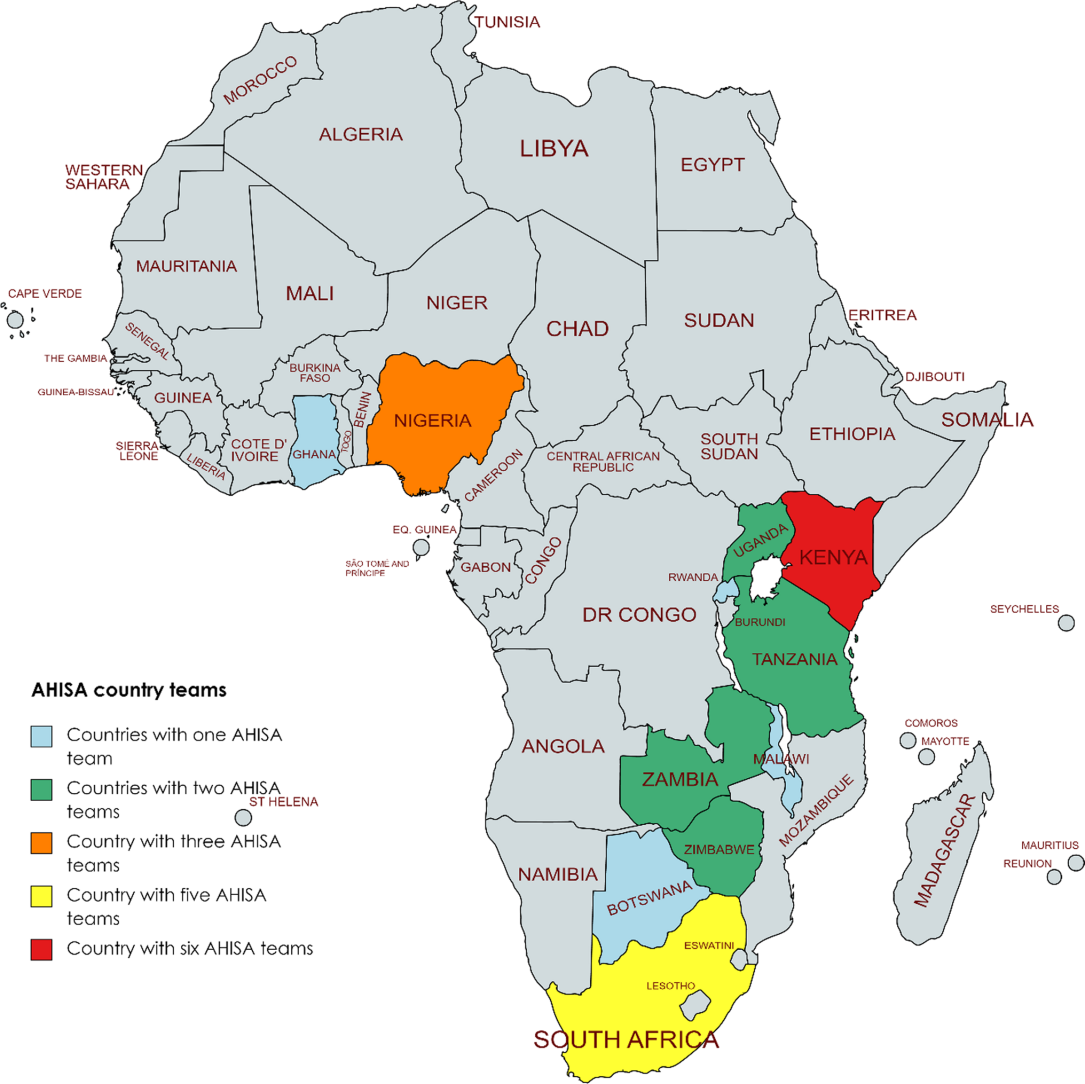
Location of AHISA teams represented in results

Two AHISA teams with overlapping membership submitted a single survey, reducing the denominator to 25 teams. Survey responses were obtained from 17 (68%) distinct AHISA teams representing 9 of the 11 (82%) AHISA countries (See Fig. 1). Teams from Uganda and Zimbabwe did not respond. Responses from 16 teams are included in this report because one team reported not having any ongoing AYA HIV prevention and/or treatment related research at the time of the pandemic. Responses to closed-ended questions related to COVID-19 pandemic impacts on study implementation reported by AHISA teams are outlined in Table 1.

**Table 1:** COVID-19 pandemic impacts on Study Implementation reported by AHISA Teams

	Number (%)
Research protocol modifications and IRB amendments required	14 (88)
Introduction of remote research activities	14 (88)
Interruptions in research recruitment	11 (85)^a^
Interruptions in research follow-up	12 (86)^b^
Study funding threatened by the COVID-19 pandemic^c^	5 (31)

^a^N = 13 for this response because some studies completed recruitment or had not yet initiated recruitment at the onset of the pandemic

^b^N = 14 for this response. Some studies consisted of only a single visit and others had completed follow-up at the onset of the pandemic

^c^Specified funding threats included needing to return funds due to inability to conduct planned group intervention, needing to pay staff despite inability to continue study activities, and inability to apply for new funding due to investigators’ COVID-specific clinical responsibilities and additional childcare responsibilities limiting time available for research

### COVID-19 Impacts on AHISA Teams’ AYA HIV Study Implementation

A key impact on the AYA HIV prevention and treatment research reported by AHISA teams included changes to study protocols and procedures. These changes were determined through collaborative decisions made by research teams and other stakeholders, including implementing partners, external advisory committees, youth advisory boards, statisticians, and funders. Examples of the COVID-19-related AYA HIV research protocol modifications described by AHISA teams are summarized in Table 2. Teams reported the drivers for these changes as being national government restrictions (e.g. lockdowns and restrictions on movement/staff working from home), limitations of facility-based care to only critical in-person services, and national medical research bodies calling for a halt of research. In one country without lockdowns and restrictions, “COVID denialism” at the national level presented unique challenges, as research and clinical teams needed to adapt safety protocols in the context of higher levels of public uncertainty.

**Table 2:** COVID-19-related AYA HIV research study protocol modifications reported by AHISA Teams

Introduction of safety plans and monitoring for coronavirus prevention • Consent updated to highlight potential risk of acquiring COVID-19 from visiting a health facility • Acquisition and utilization of masks, hand sanitizer, protective screens, and disinfectants • Tracking of COVID Impacts • Addition of COVID questions to follow-up surveys with adolescents and providers • Addition of study visit immediately after COVID-related study stoppage to assess how study stoppage may have impacted outcomes • Introduction of continuous quality improvement cycles with healthcare providers by phone
Adapted research activities to remote formats • Adapted research activities to online formats • Established security procedures for online communications • E-consent and assent • Intervention meetings conducted online • Adapted research activities to phone formats • Phone-based pre-screening of potential study participants • Introduction of phone-based consent and assent • Phone delivery of (revised) intervention tools • Phone interviews for study data collection • Phone check-ins • HIV testing location from clinic to home-based • Online study team meetings
Enrollment sites changed or closed • Utilization of schools on weekends instead of weekdays to allow for greater social distancing for adolescent recruitment • Utilization of new community-based sites • Usual study sites closed due to their being repurposed as COVID-isolation centers
Enrollment and follow-up procedures adapted • Staggered arrival times for research participants • Focus groups conducted virtually • More groups added, each with fewer participants per group to allow for social distancing

Key impacts on the AYA HIV prevention and treatment implementation research reported by AHISA teams included interruptions in research recruitment and follow-up, movement of research to remote formats, delays in study execution, reductions in enrolment, decreased frequency of follow-up, and early cessation of study recruitment prior to achieving the planned sample size. While changes that allowed for remote research activities were commonly reported, not all teams were successful in implementing remote adaptations of their study procedures. For example, one team reported not being able to convene study-related focus groups in a productive way due to social distancing requirements. Changes in clinical care also impacted important study endpoints such as viral suppression monitoring. Implementation efforts that were being studies were also disrupted with changes occurring to supports such as those aiming to improve linkage to care through in-person supports. Retention in care and care transitions also underwent pandemic-specific changes which may have been more impactful than interventions being studied in a time period that extend from the pre-pandemic to the pandemic period. For sites reporting interruptions in research recruitment and follow-up, the interruption duration was between 2 and 11 months, with one team reporting experiencing an ongoing interruption at the time of the survey.

### Young Age and Vulnerability of AYA

The young age and vulnerability of the individuals studied by AHISA team members did play into study and clinical care participation during the COVID-19 pandemic. Economic challenges which influenced the ability of some AYA to be involved in studies and clinical care were increased due to reductions of income generating opportunities for the AYA and their family members as well as COVID-19 illnesses and deaths of family wage-earners. One team reported that enrollment for children and adolescents “dropped to near zero” as travel restrictions within the country kept younger patients from being able to come to their study site. With study switches to remote formats, some AYA’s were also marginalized due to not having their own personal phones or regular access to adult caregivers’ phones. Systems that had previously been put into place, such as youth-friendly clinic accommodations, were halted during the pandemic. Some sites reported halting of disclosure support and other AYA counseling, reductions in monitoring, and lengthening of refill intervals for unstable AYA patients.

School settings provided unique challenges and opportunities for AYA HIV research during COVID-19. One team recruiting within schools reported that weekday recruitment at schools was no longer possible when schools switched to a split session (half of students attending in the mornings and half in the afternoons) to allow for greater social distancing. Other sites shared that pandemic-related school closures and altered school schedules also impacted some AYA studies in positive ways. Some sites reported that youth leaders were able to become more involved in clinic and study activities when their “bandwidth” increased due to school closures. Some sites were able to leverage the increased availability of youth leaders to increase involvement of peers in AYA engagement. At one site, AYA involvement was deemed to be essential in creation of a novel HIV self-testing confirmation system for individuals with limited internet and smartphone availability.

### Top Impacts of the COVID-19 Pandemic on AHISA Teams’ AYA HIV Research

When asked to indicate the “Top 3” impacts of the COVID-19 pandemic on their research, almost all teams indicated that the pandemic caused an extension of research timelines. Pandemic-necessitated modifications to research procedures were delayed for many teams due to slow responses from ethical bodies. Even with pandemic-specific adaptations in place, study recruitment and enrollment challenges were common. Staffing changes and increased work and life stresses facing staff, along with financial challenges, were also highlighted as key impacts of the pandemic on research progress. Some research staff were reallocated to work directly with the COVID response, while remaining staff were subject to increased stresses related to understaffing, the threat of COVID acquisition, and “operating under uncertainty.” Staff morale was a major concern at some sites. Some funding was also reallocated to urgent public health needs. In one instance, study delays impacted the team’s ability to meet a study objective that was necessary for continued funding. At the time of the survey, three teams reported having applied for supplemental funds to complete research projects with budgets that were depleted due to pandemic-related cost increases.

### Challenges to Research Endpoints and Study Validity

Impacts on research endpoints were common. COVID-19 mitigation efforts frequently altered study endpoints by lengthening the interval between clinic visits, therefore impacting studies measuring outcomes that rely on information collected during routine clinic visits. Teams indicated that rates of viral suppression, linkage to care, retention, and transition would be directly impacted by the pandemic in ways that would make it difficult to discern the impact of study interventions in studies without time-matched control groups. With youth coming to the clinic less frequently during the pandemic and moving back to ancestral homes when income-generating activities became more limited near clinic sites, there was an increase in loss to follow-up from studies. Table 3 summarizes some of the potential impacts of these changes, as well as other study design modifications and challenges owing to the COVID-19 pandemic identified within the AHISA network on study validity.

**Table 3:** Design modifications and challenges to study validity due to the COVID-19 pandemic identified within the AHISA network

Design changes and challenges	Examples from AYA HIV studies conducted among AHISA network members	Potential solutions and innovations
Under-powered studies	• Enrollment stopped early • Inability to meet recruitment goals • Increased loss to follow-up • Need for additional funding to complete aims	• Planning of new studies relevant to rapidly altered realities
Selection bias	• Fewer adolescents able to come to clinic to enroll in studies • Retention in studies more difficult for participants with fewer resources	• Investigate differences between those who do and do not enroll • Develop novel recruitment and retention strategies
Misclassification bias	• Potential for ALHIV to be classified as non-adherent to medications or clinic follow-up if they moved back to ancestral villages due to pandemic	• Tracing of AYA who are lost to follow-up
Confounding^a^	• Financial impacts of pandemic • Pandemic-related social stressors • Availability of technology	• Measurement of impacts • Measurement of access to technologic innovations
Effect modification^b^	• Availability of technology	• Measurement of access to technologic innovations
Missing data	• Unable to perform planned assessments • Missed study visits • Reduced capacity for follow-up labs • Increased loss to follow-up	• Statistical expertise to guide missing data issues • Tracing AYA who are lost to follow- up • Increasing use of qualitative methods to understand impacts
Reduced fidelity of interventions	• Loss of trained study staff • Inability to conduct study interventions in person • Rapid adaptations of interventions to remote formats	• Creation of materials to guide task- shifting • Testing and optimization of interventions adapted remote formats
Challenges to Generalizability	• Clients reluctant to interact with peer navigators as they did pre-COVID • Fewer people participated in studies during the pandemic (those who were able to participate are likely different from those who were not)	• Adapted intervention formats and decentralization of care to increase access and generalizability for marginalized populations

^a^Factors that may be measured or unmeasured within studies associated with both exposures and outcomes, uncontrolled will bias the effect estimate calculated

^b^Magnitude of an effect estimate varies between groups when assessing the relationship between an exposure and an outcome; when anticipated should be factored into sample size calculations

### Lessons Learned

Some AHISA teams noted that they did not yet feel that they had helpful lessons to share. These teams were still in “crisis mode,” not yet having learned what might help them overcome challenges wrought by the pandemic. However, others reported that the pandemic taught them important lessons and forced them to make changes that are likely to have long-lasting impacts on both AYA HIV-related care and their implementation research. Key lessons learned for AYA HIV-Related Research are highlighted in Box 1.

**Table:** **Box 1** Key Lessons Learned for AYA HIV-Related Research

AYA can demonstrate remarkable resilience in the face of rapid changes in their needs and environment
Remote service delivery can improve access for AYA, although some AYA remain marginalized by limited technology
Readiness to innovate improves responsiveness to change
Compassion and flexibility are important for both AYA and staff wellness

Although all AHISA teams are experienced in working with AYA, one of the key reflections mentioned by teams was renewed appreciation for the resilience of AYA. One emphasized that we must “learn from young people to adapt strategies to meet their dynamic needs and environment.” The pandemic underscored the need to continuously engage with key implementers and stakeholders, particularly the AYA themselves. Some teams also stressed the need to “pivot” their activities to meet the most pressing needs of the populations they serve, even when that meant pausing research and drawing resources towards the populations’ basic needs. For example, with economic challenges increased for the youth being served by AHISA teams, one research team worked with a non-governmental organization (NGO) to provide care packages with food, basic necessities (e.g. masks, soap), and transport reimbursement to the clinic to encourage ALHIV to come for medication refills.

Although expressed with caveats, several teams noted learning that “remote delivery of services works.” For many, the pandemic created the opportunity and necessity to increase use of technology, both for research and for service implementation. One team noted with surprise that holding study workshops virtually led to better attendance than in-person meetings. Another team stressed that some adolescents were more willing to open-up and share when on the phone compared with in-person encounters, allowing healthcare providers to provide better care. This team suggested that even outside the context of the pandemic, phone delivery might be a good alternative strategy for improving relationships with ALHIV. However, teams also learned that there was a need for flexibility in timing of calls in order to reach AYA, and a need to provide training and support for providers newly delivering remote services.

Having strategies and processes in place for planning and making adaptations, when necessary, were stressed as key influences on being ready to innovate when the pandemic first started to affect research studies. One team shared the lesson that “implementation science should be adjustable for changes. However, these changes and events should be well documented to allow for contextual data interpretation.” The unprecedented rapid changes brought forth by the pandemic made these needs particularly salient. Providing more specific guidance for strategies for coordinated adaptation, another team suggested that “team members should be trained and have access to multiple communication platforms to ensure connectivity is maintained as much as possible.” Some teams faced strict lockdowns, combined with power outages that impacted electricity supply, internet connectivity, and cell phone tower reception. Having study processes in place to ensure that devices and backup power banks were kept fully charged and that study cell phones included internet data for hot spot internet connections to other research devices was found to be helpful. Expanding the research capacity of implementation partners was an unexpected strategy when some research sites were converted to service COVID-related public health demands. Stakeholders who value the work being done by these implementation research teams were invested in helping teams meet the challenges of continuing research during a global pandemic.

Finally, several teams expressed learning or being reminded of the need for compassion and flexibility in the face of difficult times. One team noted “it is vital to maintain a supportive and understanding attitude towards colleagues as the pandemic had sometimes significant negative effects on staff including both mental and physical aspects.” For both staff and the AYA they serve, “many had family members die, obviously affecting the social dynamics.” Recognizing the humanity in co-workers, there was increased need for “flexibility, creativity, patience, and willingness to think out of the box.”

## Discussion

This survey showed that COVID-19 has impacted AYA HIV implementation research within the AHISA network in both negative and positive ways. This included challenges to both the completion and the validity of ongoing research, coupled with innovations that are likely to accelerate the field. While most pandemic impacts have been negative and AYA, especially ALHIV are likely disproportionately impacted, some positive impacts such as development of new remote service delivery models are likely to reap long-lasting benefits for marginalized and difficult-to-reach AYA populations as well as healthcare and research teams. The findings of this survey suggest that we may better prepare ourselves as researchers to adapt to unexpected challenges by increasing the substantive involvement of the AYA in research and innovation-development, by investing in the development and testing of remote service delivery models, by maintaining adaptable systems, and by increasing our capacity for compassion and flexibility.

While there is controversy regarding whether COVID-19 infection itself is appreciably more severe among PLHIV [39, 40], it is clear that this population group bears a disproportionately higher burden of structural vulnerabilities that have been increased further by the pandemic [8]. These vulnerabilities are impacting both HIV-related outcomes and research aiming to improve those outcomes. Published suggestions for strategies to ensure that HIV research continues amidst the challenges brought by the COVID-19 pandemic have included use of social media and network-based recruiting, online prescreening forms with a follow-up call, intervention delivery through telehealth and text messaging, and laboratory samples collected in home settings [41, 42]. Our data shows that many of these strategies are being employed among teams conducting HIV prevention and treatment research among AYA in Africa through the AHISA network. AHISA teams highlighted that some of these strategies have unique advantages for segments of this target population such as improved engagement of some AYA using remote platforms compared to in-person strategies. However, since AYA in Africa frequently lack access to mobile phones [43, 44], reliance on mobile phone-based interventions may increase inequalities.

AHISA teams completed surveys before April 2021. Most participating countries have experienced additional “waves” of COVID-19 which have necessitated further lockdowns and restrictions that were not captured through this survey [36, 37]. The elapsed time since the surveys were conducted has allowed teams to further adapt to the challenges of conducting research during a pandemic, including some healthcare worker access to vaccinations [38]. However, additional waves of infection within countries have also further challenged systems, further extended research timelines, and created new funding difficulties. With the slow pace of COVID-19 vaccination roll out in Africa [38] and the increasing presence of COVID-19 variants, ongoing challenges will force us to continue to adapt to carry out essential AYA HIV-related implementation research.

Strengths of this manuscript include the utilization of a strong, relatively well-funded network of AYA-focused researchers in Africa who together can fill a current gap in knowledge related to COVID-19 pandemic-related impacts on AYA HIV implementation research. Our sample includes a good representation of AYA HIV studies in the field, although it is not exhaustive. Limitations include the fact that the survey was conducted at only a single timepoint in the evolution of the COVID-19 pandemic. Furthermore, the sample is specific to Africa and generalizability to AYA research in other settings may be limited. AHISA teams all include NIH-funded implementation researchers. Smaller and less well-funded teams lacking NIH-funded partners may have unique issues that were likely underreported.

With AYA, especially those in high HIV-burden African countries, being uniquely vulnerable to HIV infection and to poor HIV outcomes [29, 45], it is urgent that research focused on improving outcomes in this population is accelerated. Results of our survey show that to the contrary, research focused on AYA living with and at risk of contracting HIV in high burden settings is likely being disproportionately impacted by the COVID-19 pandemic. While the threat of COVID-19 to implementation science studies is clear, there is also a clear opportunity to apply implementation science principles and tools in adapting to pandemic disruptions while conducting HIV implementation research focused on AYA. By actively engaging the context in which pandemic-related disruptions and innovations are taking place, we can more rapidly move towards increased uptake and sustainability of strategies that improve care for AYA across the HIV continuum.

## Conclusion

Implementation science studies conducted by AHISA teams in African countries have been strongly impacted by the COVID-19 pandemic. Most of the impacts have been negative and risk compromising progress in closing knowledge-action-outcomes gaps. However, some positive adaptations have emerged such as the leveraging of technology for virtual/remote service delivery and rapid decentralization of care. Implementation science can be further applied to guide, strengthen, and formalize these adaptations for potential long-term benefits.

## Supplementary Information

Below is the link to the electronic supplementary material.

Supplementary file1 (PDF 628 KB)
