# Supplementary material for: Impact of COVID-19 on Adolescent HIV Prevention and Treatment Research in the AHISA Network
Source: AIDS Behav. 2022 Sep 12;27(Suppl 1):73–83. doi: 10.1007/s10461-022-03811-5 (PMC9466311; doi:10.1007/s10461-022-03811-5)
Supplement: Supplementary file 1 — Supplementary file1 (PDF 628 KB) [file 10461_2022_3811_MOESM1_ESM.pdf]

AHISA COVID Survey

SURVEY INSTRUCTIONS.

Thank you for agreeing to complete a survey of how your AHISA team and partner sites have been impacted by the COVID-19 pandemic!

The **purposes** of this survey are:

- To assess what AHISA teams adolescent HIV prevention and treatment programming (clinical and/or support services") is being impacted by the COVID-19 pandemic
- To assess how AHISA teams' adolescent HIV prevention and treatment research is being impacted by the COVID-19 pandemic
- To compile information regarding what teams are learning and how they are adjusting during the COVID-19 pandemic.

We ask that each AHISA team complete one survey **by February 10, 2021**. An electronic copy of the survey has been provided to all AHISA team members to facilitate conversations with key local stakeholders needed to accurately complete the survey. **An online survey link was provided to the AHISA team PI (or another designated team member) for completion of a single survey on behalf of each AHISA team .**

Because we know that some AHISA teams are involved in many projects, we are asking you to answer the survey specifically about:

1. Two clinical or support services*
2. Two research projects*

The **two chosen services and projects** should be selected by the team as representing:

- Clinical services and research projects that you think represent the ***best examples of effective responses to the disruptions threatened by the COVID-19 pandemic***
- Clinical services and research projects that you think have been ***most negatively affected by the COVID-19 pandemic***

*One clinical or support service and one research project can be discussed if a single example represents BOTH what was most negatively affected and the best example of an effective response. e.g. a study that was completely shut down due to COVID then implemented a creative solution to restart and make up for lost enrollment time.

This information will be used to compile a summary of experiences and "lessons learned" which will be both disseminated back to the AHISA teams in aggregate and written up for potential publication in a peer-reviewed journal.

For this survey, please consider Adolescent HIV Prevention and Treatment services broadly, including HIV testing, PrEP, STD treatment, sexual and reproductive health, linkage to care and/or monitoring, HIV treatment, viral suppression monitoring, retention-support services and/or monitoring, adolescent transition of care services and/or monitoring, HIV support groups, HIV-related services, other forms of psychosocial support. Similarly, research in any of these areas is relevant to the purposes of this survey.

It should take between 30 minutes and 1 hour to complete the on-line survey after your team agrees regarding the best examples to share. We thank you very much in advance for your time and for sharing your experiences!

If you must save and return to the survey at a later time please see the steps below.

PLEASE NOTE: you will need the RETURN CODE to continue your survey. If you select to have the survey link emailed to you *the return code is NOT included in the email*. Please make sure to write it down before closing the survey.

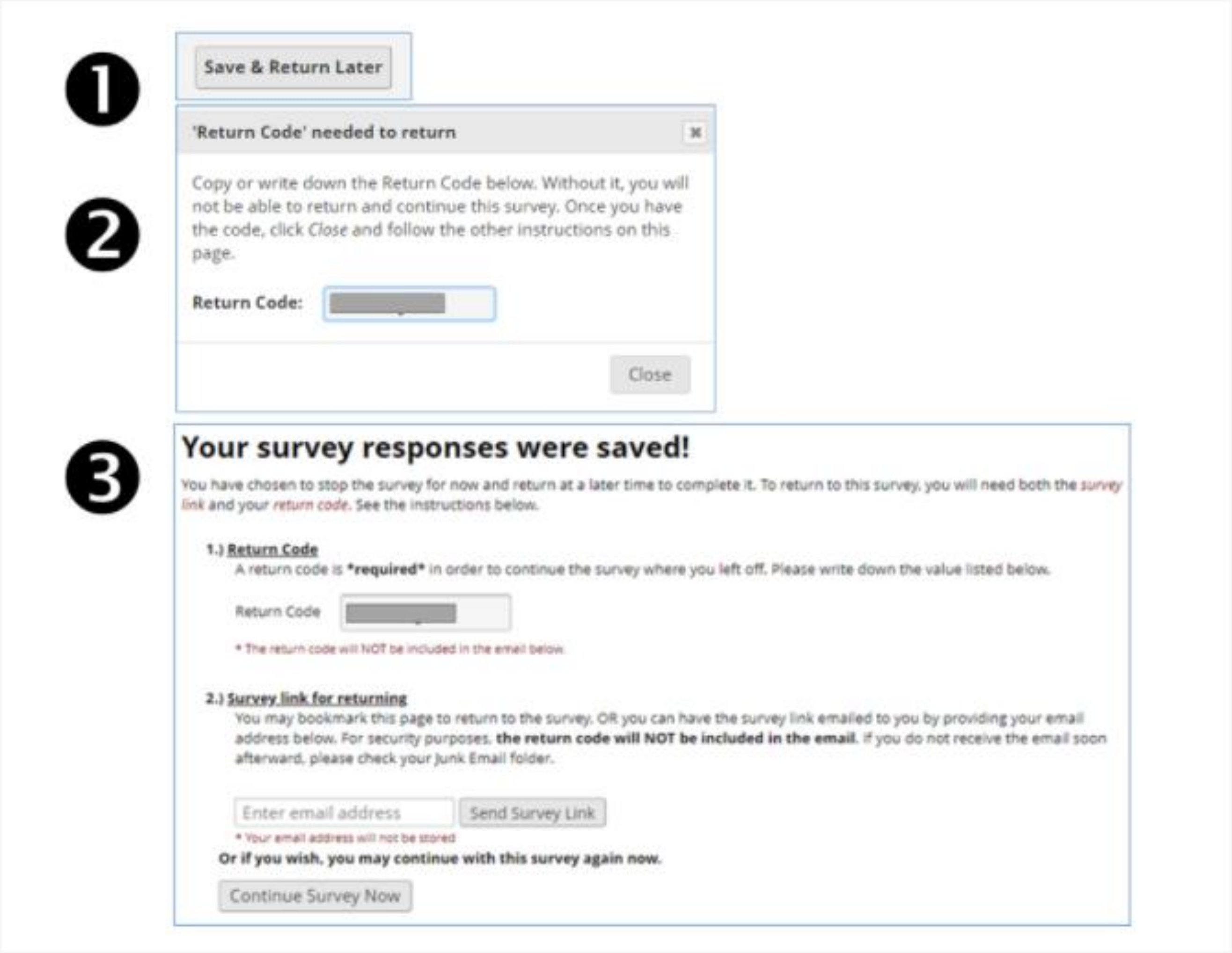

Next Page >>

SECTION 1.

SITE & SITE CONTACT INFORMATION.

Country represented by your AHISA team:

* must provide value

- Botswana
- Ghana
- Kenya
- Malawi
- Nigeria
- Rwanda
- South Africa
- Tanzania
- Uganda
- Zambia
- Zimbabwe
- Other

SITE CONTACT: first name | last name

Please select the best way(s) to contact you *and* include your contact information.

- **Email** email address
- **Whatsapp** Whatsapp info
- **Skype** Skype info
- **Other** Other contact info

<< Previous Page | Next Page >>

SECTION 2.

Part 1. Identification & Details of **Clinical Service** Examples

Please provide a title and brief description of the adolescent-focused HIV prevention and/or treatment *clinical service* your team has identified as the best example of effective responses to the disruptions threatened by the COVID-19 pandemic.

Please provide a title and brief description of the adolescent-focused HIV prevention and/or treatment *clinical service* your team has identified as the most negatively affected by the COVID-19 pandemic.

Is the adolescent-focused HIV prevention and/or treatment *clinical service* that was most negatively affected by the COVID-19 pandemic THE SAME as the best example of an effective response?

Yes | No

reset

Clinical Service Example #1
Title:

Clinical Service Example #1.
Description.

Please include the location of the service (e.g. nationwide, multi-site but not national, single referral center, community-based), the nature of the service (e.g. testing, treatment, support group).

Expand

Who or what directed the change?

Expand

Please describe in as much detail as possible what was changed to effectively adapt to potential negative impacts of the COVID-19 pandemic.

Please include a description of what populations were most affected. Please describe changes that occurred at the policy-level, the facility-level, the patient-level, and the community-level if relevant.

Expand

Clinical Service Example #2
Title:

If different from "Clinical Service Example #1"

Clinical Service Example #2.
Description.

Please include the location of the service (e.g. nationwide, multi-site but not national, single referral center, community-based), the nature of the service (e.g. testing, treatment, support group).

If different from "Clinical Service Example #1" (effective response)

Expand

Who or what directed the change?

Expand

Please describe in as much detail as possible what negative impacts were experienced as a result of the COVID-19 pandemic.

Please include a description of what changed and what populations were most affected.

Expand

<< Previous Page | Next Page >>

SECTION 2.

Part 2. Identification & Details of **Research Project** Examples

Please provide a title and brief description of the adolescent-focused HIV prevention and/or treatment *research* your team has identified as the best example of effective responses to the disruptions threatened by the COVID-19 pandemic.

Please provide a title and brief description of the adolescent-focused HIV prevention and/or treatment *research* your team has identified as the most negatively affected by the COVID-19 pandemic.

Is the adolescent-focused HIV prevention and/or treatment *research* that was most negatively affected by the COVID-19 pandemic THE SAME as the best example of an effective response?

Yes

No

reset

Research Example #1

Title:

Research Example #2

Title:

if different from "Research Example #1"

Research Example #1.
Description.

Please include the location of the research (e.g. nationwide, multi-site but not national, single referral center, community-based), the nature of the research including the topic(s) being addressed (e.g. testing, treatment, support group), and the study design (e.g. clinical trial, prospective cohort).

Expand

Research Example #2.
Description.

Please include the location of the research (e.g. nationwide, multi-site but not national, single referral center, community-based), the nature of the research including the topic(s) being addressed (e.g. testing, treatment, support group), and the study design (e.g. clinical trial, prospective cohort).

if different from "Research Example #1" (effective response)

Expand

Who or what directed the change?

Expand

Who or what directed the change?

Expand

Please describe in as much detail as possible what was changed to effectively adapt to potential negative impacts of the COVID-19 pandemic.

Please include a description of what populations were most affected. Please describe changes that occurred at the policy-level, the facility-level, the patient-level, and the community-level if relevant.

Expand

Please describe in as much detail as possible what negative impacts were experienced as a result of the COVID-19 pandemic.

Please include a description of what changed and what populations were most affected.

Expand

<< Previous Page

Next Page >>

Section 3.

Open-Ended Assessments of Impact

Please describe what you think are the three most important impacts of the COVID-19 pandemic on *clinical services* for adolescents at your site(s):

IMPORTANT IMPACT #1.	IMPORTANT IMPACT #2.	IMPORTANT IMPACT #3.
Expand	Expand	Expand

Please describe what you think are the three most important impacts of the COVID-19 pandemic on adolescent-focused *research* at your site(s):

RESEARCH IMPACT #1.	RESEARCH IMPACT #2.	RESEARCH IMPACT #3.
Expand	Expand	Expand

Please describe the 3 most important things your team(s) has/have adjusted or changed due to the COVID-19 pandemic.

CHANGE #1.	CHANGE #2.	CHANGE #3.
Expand	Expand	Expand

Please describe any lessons that your team has learned that you think may be helpful for other teams in the AHISA network.

Expand

<< Previous Page

Next Page >>

Section 4.

In the following section, please select the box next to the best answer to indicate if each of the following has been experienced at your site or sites as a result of the COVID-19 pandemic. For this section, please consider services and research in the country or region represented by your AHISA team overall, *not exclusively those given as examples in Sections 1 and 2.*

For answers indicating a change due to the COVID-19 pandemic, please provide as much detail as possible in the space provided.

Have there have been periods of time when certain adolescent HIV prevention and treatment services were unavailable due to the COVID-19 pandemic?

- Yes
- No
- Not applicable

reset

If yes, which services were unavailable?

leave blank if no service disruptions

Expand

If yes, what were the approximate date(s) and/or duration of service disruption(s):

leave blank if no service disruptions

Expand

Since the beginning of the COVID-19 pandemic, access to adolescent sexual and reproductive health services has:

- Increased
- Decreased
- Remained the same
- Not applicable

reset

Please provide additional detail here:

optional

Expand

Since the beginning of the COVID-19 pandemic, adolescent access to PrEP has:

- Increased
- Decreased
- Remained the same
- Not applicable

reset

Please provide additional detail here:

optional

Expand

Based on our clinical experience and/or available data, since the beginning of the COVID-19 pandemic, adolescent HIV testing has:

- Increased
- Decreased
- Remained the same
- Not applicable

reset

Please provide additional detail here:

optional

Expand

Based on our clinical experience and/or available data, since the beginning of the COVID-19 pandemic, adolescent HIV incidence has:

- Increased
- Decreased
- Remained the same
- Not applicable

reset

Please provide additional detail here:

optional

Expand

Based on our clinical experience and/or available data, since the beginning of the COVID-19 pandemic, adolescent sexually-transmitted infections (other than HIV) incidence has:

- Increased
- Decreased
- Remained the same
- Not applicable

reset

Please provide additional detail here:

optional

Expand

Based on our clinical experience and/or available data, since the beginning of the COVID-19 pandemic, adolescent linkage to HIV care has:

- Increased
- Decreased
- Remained the same
- Not applicable

reset

Please provide additional detail here:

optional

Expand

Since the beginning of the COVID-19 pandemic, the number of patients missing scheduled clinical visits has:

- Increased
- Decreased
- Remained the same
- Not applicable

reset

If applicable, please give us your top-3 reasons for this change in patients missing clinical visits

Reason #1

Reason #2

Reason #3

If applicable, how have these changes in patient missed clinical visits impacted patient safety?:

Expand

Based on our clinical experience and/or available data, since the beginning of the COVID-19 pandemic, have any ART tracer drugs experienced stockouts?

- yes
- no
- unsure

reset

Please provide additional detail here:

optional

Expand

Since the beginning of the COVID-19 pandemic, the duration between prescription refills has:

- Increased
- Decreased
- Remained the same
- Not applicable

reset

Please provide additional detail here:

optional

Expand

Since the beginning of the COVID-19 pandemic, the scheduled duration between routine monitoring visits has:

- Increased
- Decreased
- Remained the same
- Not applicable

reset

Please provide additional detail here:

optional

Expand

Based on our clinical experience and/or available data, since the beginning of the COVID-19 pandemic, rates of HIV treatment adherence have:

- Increased
- Decreased
- Remained the same
- Not applicable

reset

If applicable, please give us your top-3 reasons for this change in treatment adherence rates

Reason #1

Reason #2

Reason #3

Since the beginning of the COVID-19 pandemic, availability of adolescent HIV-related laboratory monitoring has:

- Increased
- Decreased
- Remained the same
- Not applicable

reset

Please provide additional detail here:

optional

Expand

Based on our clinical experience and/or available data, since the beginning of the COVID-19 pandemic, rates of adolescent HIV virologic suppression have:

- Increased
- Decreased
- Remained the same
- Not applicable

reset

Please provide additional detail here:

optional

Expand

Since the beginning of the COVID-19 pandemic, the need for adolescent psychosocial support service has:

- Increased
- Decreased
- Remained the same
- Not applicable

reset

Please provide additional detail here:

optional

Expand

Since the beginning of the COVID-19 pandemic, the availability of adolescent psychosocial support services has:

- Increased
- Decreased
- Remained the same
- Not applicable

reset

Please provide additional detail here:

optional

Expand

Since the beginning of the COVID-19 pandemic, peer-to-peer support for adolescents has: *(select all that apply)*

- maintained old models
- added an electronic support system
- transitioned completely to an electronic support system
- been interrupted
- been increased

Based on our clinical experience and/or available data, since the beginning of the COVID-19 pandemic, adolescent mental health emergencies have:

- Increased
- Decreased
- Remained the same
- Not applicable

reset

Please provide additional detail here:

optional

Expand

Based on our clinical experience and/or available data, since the beginning of the COVID-19 pandemic, adolescent pregnancy rates have:

- Increased
- Decreased
- Remained the same
- Not applicable

reset

Please provide additional detail here:

optional

Expand

Since the beginning of the COVID-19 pandemic, programmatic funding related to adolescent HIV prevention and treatment has:

- Increased
- Decreased
- Remained the same
- Not applicable

reset

Please provide additional detail here:

optional

Expand

Since the beginning of the COVID-19 pandemic, how many of your clinical staff have been diagnosed with definite or probable SARS-CoV-2 infection?

(if none, enter 0)

- I do not know / Prefer not to answer

Clinical staff roles of those diagnosed with SARS-CoV-2 (check all that apply)

- Doctors
- Nurses
- Pharmacy Staff
- Other (if other, please specify)

Since the beginning of the COVID-19 pandemic, please indicate how many of your adolescent patients were diagnosed with definite or probable SARS-CoV-2 infections:

(if none, enter 0)

- I do not know / Prefer not to answer

<< Previous Page

Next Page >>

Research

Since the beginning of the COVID-19 pandemic, have there been times when study recruitment was halted *due to the COVID-19 pandemic?*

- Yes
- No
- Not applicable

reset

Please provide details of study recruitment stoppage(s).

leave blank if no study recrutment disruptions

Expand

If yes, what were the approximate dates and/or length of recruitment stoppage(s):

leave blank if no study recrutment disruptions

Expand

Since the beginning of the COVID-19 pandemic, have there been times when study follow-up was halted *due to the COVID-19 pandemic?*

- Yes
- No
- Not applicable

reset

Please provide details of study follow-up stoppage(s).

leave blank if no study follow-up disruptions

Expand

If yes, what were the approximate dates and/or length of follow-up stoppage(s):

leave blank if no study follow-up disruptions

Expand

Since the beginning of the COVID-19 pandemic, has the enrollment rate(s) for your studies:

- Increased
- Decreased
- Remained the same
- Not applicable

reset

Please provide additional detail here:

Expand

Based on our clinical experience and/or available data, since the beginning of the COVID-19 pandemic, the number of patients missing scheduled study visits has:

- Increased
- Decreased
- Remained the same
- Not applicable

reset

If applicable, please give us your top-3 reasons for this change in missed study visits

Reason #1

Reason #2

Reason #3

If applicable, how have these changes in missed study visits impacted patient safety?:

Expand

Since the beginning of the COVID-19 pandemic, were protocol modifications/new IRB approvals needed due to COVID-19 related procedural changes?

- Yes
- No
- Not applicable

reset

Please provide additional detail here:

Expand

Since the beginning of the COVID-19 pandemic, were any research activities changed from in-person to remote formats?

- Yes
- No
- Not applicable

reset

Please provide additional detail here:

Expand

Since the beginning of the COVID-19 pandemic, have you needed to apply for supplemental funding to complete previously funded research due to pandemic-related cost increases?

- Yes
- No
- Not applicable

reset

Please provide additional detail here:

optional

Expand

Has your study funding been threatened by the COVID-19 pandemic?

Yes

No

Not applicable

reset

Please provide additional detail here:

optional

Expand

Since the beginning of the COVID-19 pandemic, how many of your research staff have been diagnosed with definite or probable SARS-CoV-2 infection?

(if none, enter 0)

I do not know / Prefer not to answer

Research staff roles of those diagnosed with SARS-CoV-2 (check all that apply)

Doctors

Nurses

Pharmacy Staff

Other (please specify)

Since the beginning of the COVID-19 pandemic, please indicate how many of your research study patients were diagnosed with definite or probable SARS-CoV-2 infections:

(if none, enter 0)

I do not know / Prefer not to answer

<< Previous Page

Submit
